# Identification of Markers for Diagnosis and Treatment of Diabetic Kidney Disease Based on the Ferroptosis and Immune

**DOI:** 10.1155/2022/9957172

**Published:** 2022-11-23

**Authors:** JingYuan Ma, ChangYan Li, Tao Liu, Le Zhang, XiaoLing Wen, XiaoLing Liu, WenXing Fan

**Affiliations:** ^1^Department of Nephrology, The First Affiliated Hospital of Kunming Medical University, Kunming, Yunnan 650032, China; ^2^Organ Transplantation Center, The First Affiliated Hospital of Kunming Medical University, Kunming, Yunnan 650032, China; ^3^Institute for Integrative Genome Biology, University of California Riverside, Riverside, California 92521, USA; ^4^Kunming Medical University, Kunming, Yunnan 650500, China

## Abstract

**Background:**

In advanced diabetic kidney disease (DKD), iron metabolism and immune dysregulation are abnormal, but the correlation is not clear. Therefore, we aim to explore the potential mechanism of ferroptosis-related genes in DKD and their relationship with immune inflammatory response and to identify new diagnostic biomarkers to help treat and diagnose DKD.

**Methods:**

Download data from gene expression omnibus (GEO) database and FerrDb database, and construct random forest tree (RF) and support vector machine (SVM) model to screen hub ferroptosis genes (DE-FRGs). We used consistent unsupervised consensus clustering to cluster DKD samples, and enrichment analysis was performed by Gene Set Variation Analysis (GSVA), Gene Ontology (GO), and Kyoto Encyclopedia of Genes and Genomes (KEGG) and then assessed immune cell infiltration abundance using the single-sample gene set enrichment analysis (ssGSEA) and CIBERSORT algorithms. Ferroptosis scoring system was established based on the Boruta algorithm, and then, core compounds were screened, and binding sites were predicted by Coremine Medical database.

**Results:**

We finally established a 7-gene signature (DUSP1, PRDX6, PEBP1, ZFP36, GABARAPL1, TSC22D3, and RGS4) that exhibited good stability across different datasets. Consistent clustering analysis divided the DKD samples into two ferroptosis modification patterns. Meanwhile, autophagy and peroxisome pathways and immune-related pathways can participate in the regulation of ferroptosis modification patterns. The abundance of immune cell infiltration differs significantly across patterns. Further, molecular docking results showed that the core compound could bind to the protein encoded by the core gene.

**Conclusions:**

Our findings suggest that ferroptosis modification plays a crucial role in the diversity and complexity of the DKD immune microenvironment, and the ferroptosis score system can be used to effectively verify the relationship between ferroptosis and immune cell infiltration in DKD patients. Kaempferol and quercetin may be potential drugs to improve the immune and inflammatory mechanisms of DKD by affecting ferroptosis.

## 1. Introduction

Diabetic kidney disease (DKD) is a chronic microvascular disease. High glucose environments activate inflammatory immune responses, promote podocyte and mesangial cell damage, and reduce renal function [1]. However, the regulation of this process is still unclear. Previous studies have found that activating immune T cells, B cells, and macrophages in DKD involves some renal biological regulation processes. However, it does not yet account for the deterioration and progression of the kidneys in end-stage patients in a high-glucose setting. The current research point of view is that malignant changes such as anemia, electrolyte imbalance, and abnormal lipid metabolism in patients with end-stage renal disease are closely related to the decline of erythropoietin (EPO) and glomerular filtration capacity [2]. Lower EPO levels, especially in the presence of iron deficiency, predict a rapid loss of kidney function. A new view suggests that DKD patients have iron overload in addition to iron deficiency. Abnormal iron accumulation may increase oxidative stress and reduce antioxidant capacity, leading to the development of DKD [3]. Iron is filtered through the glomerulus and reabsorbed in the renal tubule. The renal tubular epithelial cells are active sites for iron ions and reactive oxygen species. Ferroptosis occurs when the cell is overloaded, and lipid peroxidation is increased, producing a lot of reactive oxygen species (ROS) [4–6]. Ferroptosis is distinct from necrosis, apoptosis, and autophagy in cellular morphology and function. In morphology, ferroptosis mainly occurred in cells, which showed that the volume of mitochondria decreased, the density of the bilayer membrane increased, the mitochondrial crista decreased or disappeared, but the cell membrane was intact, and the nuclear morphology did not change. Biochemically, intracellular depletion of glutathione (GSH) can lead to glutathione peroxidase 4 (GPX4) activity [7]. The reduction of GPX4, in turn, causes GPX4-catalyzed GSH to fail to metabolize lipid oxides. Then, lipids will generate many ROS after Fe+2 is oxidized in the Fenton reaction, thereby promoting ferroptosis in cells [8].

Abnormal iron metabolism can cause renal tubular dysfunction and adversely affect the maintenance of renal function. For example, Liao et al. found that iron deposition in the tubular epithelial cells of patients with DKD may produce nephrotoxicity and damage the kidneys [9]. Li et al. found that the concentrations of malondialdehyde (MDA) and 4-hydroxytryptamine (4-HNE) increased in the DKD model, and ROS increased significantly in renal cortical proximal tubule epithelial cells (HK2) under a high glucose environment. Mitochondria shrank, cell membrane density increased, and mitochondrial crystal decreased [10]. In animal studies, DKD mouse models show characteristic signs of iron death. For example, intracellular ACSL4 expression is upregulated and GPX4 and cysteine/glutamate antitransport system expression is decreased under high glucose environment, leading to decreased antioxidant capacity and increased lipid peroxide production [11]. In addition, inhibition of ferroptosis can delay the development of renal lesions in diabetic mice. Studies have shown that islets can produce excessive iron under high glucose conditions. Iron deposition can cause a Fenton reaction between free iron ions and hydrogen peroxide, destroying reactive free radicals in cells and inducing programmed ferroptosis. The above evidence suggests that abnormal iron metabolism may affect the progression of DKD, but the exact mechanism is unclear [12]. Iron overload directly or indirectly causes intracellular lipid peroxidation and impairs cell structure and function. Relevant evidence suggests that it may be associated with aberrant oxidative phosphorylation pathway in mitochondria, ATP and TRP, oxidizing cells, and polyunsaturated fatty acids (PUFA) [13]. The arachidonic acid (AA) is isolated from PUFA by enzymatic action. AA is involved in inflammation through three metabolic pathways, cyclooxygenase (COX), lipooxygenase (LOX), and CYP450 enzymes [14, 15], which further exacerbates iron drooping. DKD is associated with systemic and local kidney inflammation. The levels of inflammatory cytokines such as interleukin- (IL-) 1, IL-6, tumor necrosis factor- (TNF-) *α*, monocyte chemoattractant protein- (MCP-) 1, and macrophage inflammatory protein- (MIP-) 1*β* can be activated. Iron metabolism can regulate inflammatory cytokines in macrophages, promote inflammatory polarization through the TLR4/TRIF pathway, and induce the expression of proinflammatory cytokines [16, 17]. Kim et al. used TGF-*β*1-stimulated proximal tubular epithelial cells and diabetic mouse models to conduct in vitro and in vivo experiments. In animal studies, the expression of xCT and GPX4 mRNA in diabetes mellitus (DM) biopsies was lower than in controls. Reduced GSH concentration and enhanced lipid peroxidation in TGF-*β*1-stimulated tubular cells were associated with ferroptosis [18]. Activated T cells are in high demand for iron, and iron deficiency inhibits T cell proliferation. Iron overload leads to an imbalance between CD4 and CD8 T cells, and iron overload can also increase ROS levels in the body [19]. TGF-*β*1 secreted by macrophages regulates the expression of ferroptosis-related target genes ZEB1 and SLC7A11 by activating SMAD signaling transcription. In contrast, SLC7A11 gene deletion promotes ferritin atrophy induced by iron overload-induced ferroptosis in macrophages [20–22]. Taken together, we speculate that ferroptosis is closely related to the immune status of DKD and stimulates inflammatory factors to affect the progression of DKD.

However, the mechanisms underlying abnormal iron metabolism and immune inflammation in DKD are unclear. Therefore, further studies on the roles of ferroptosis and immune status in the pathogenesis of DKD are urgently needed to elucidate their associated signaling pathways, which can help provide new molecular targets for the treatment of DKD. In this study, we used the combined dataset to screen out the core diagnostic genes of ferroptosis and carried out internal and external validation. Then, we identified two molecular clusters related to ferroptosis modification for DKD. Then, we investigated the interaction between the two characteristic modification modes of iron death, the immune microenvironment, and the bioinformatics enrichment. After gene clustering, we further verified the relationship between the clustering effect and the immune microenvironment. To characterize and quantify each subtype, we developed a ferroptosis scoring system to validate the association of clustering effects with the abundance of immune cell infiltration. Finally, network pharmacology screens core compounds based on hub DE-FRGs and enriched immune pathways. Then, we used AutoDock Vina molecular docking software to predict the binding sites of small ligands to core genes. This way, we can explore the relationship between abnormal iron metabolism and renal injury in DKD patients and help make new clinical decisions.

## 2. Materials and Methods

### 2.1. Data Acquisition

**Figure 1:**
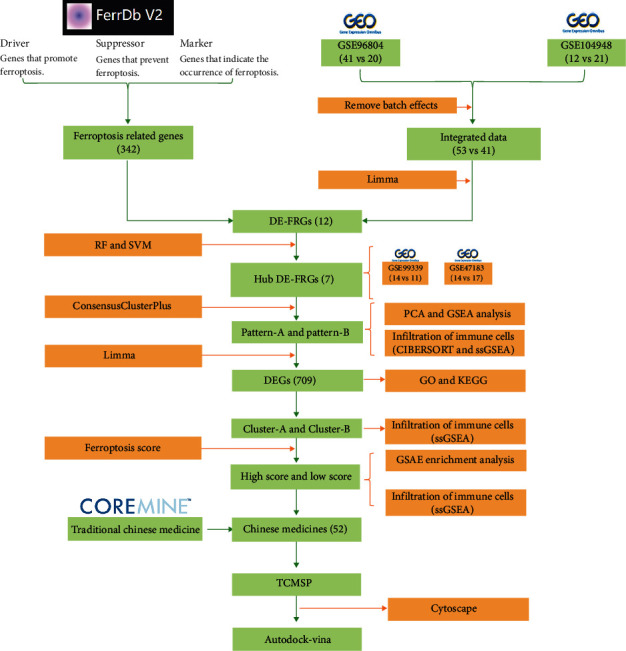
Flowchart of research design and analyzing process of this study.

From the FerrDb database (http://www.zhounan.org/ferrdb/), ferroptosis-related genes (FRGs) include 150 driver genes, 69 suppressor gene, and 123 marker genes [23]. The transcriptome expression profile datasets GSE96804, GSE104948, GSE47183, and GSE30122 were downloaded from the GEO database (https://www.ncbi.nlm.nih.gov/geo/) (Table 1). Download the dataset's microarray normalized expression matrix and annotate the probes using the dataset's annotation file. After ID conversion, the average expression value is taken as the gene expression value when multiple searches correspond to one gene. Using the ComBat algorithm of R software “SVA” package for batch correction of datasets, log2 transformation of datasets was performed, and datasets GSE96804 and GSE104948 were combined as training sets. Datasets GSE47183 and GSE30122 were used for verification sets. Thus, all data were freely available. The workflow and data preprocessing are illustrated in Figure 1.

### 2.2. Ferroptosis-Related Gene Screening

The “Limma” package of the R software extracted expression levels of iron death-related genes in the combined dataset. Differential genes were screened from DKD and CON samples and defined as the differentially expressed ferroptosis-related genes (DE-FRGs). Selection criteria are |log2FC| ≥ 1 with adjusted *p* − value < 0.05 gene as DE-FRGs. Use R software's “HeatMap” packages to create heat maps and R software “Circos” packages to draw DE-FRGs on the chromosome position map.

### 2.3. Diagnosis-Related DE-FRGs with RF and SVM

A random forest (RF) generates multiple decision trees and uses information from each tree to make predictions [24]. We used stochastic forests for feature selection and the relationship between error rates and classification trees to reveal genes of greater relative importance than 2 as final signatures. In the meantime, the expression data of DE-FRGs were applied to the machine learning algorithm of the support vector machine (SVM) classifier to obtain disease characteristic genes. The aim of SVM algorithm is to identify a decision hyperplane that makes the distance between the hyperplane and the instances that are closest to boundary is maximized. By introducing the concept of “soft margin” and using “kernel trick,” SVM performs well with linear indivisibility data [25]. The intersection of the characteristic genes obtained by the two algorithms is called the hub DE-FRGs. Estimating the area under the subject operating curve (AUC) indicated the predictive diagnostic effect. The receiver operating characteristic (ROC) analysis was performed in RStudio by the pROC package. DE-FRGs have a diagnostic value in DKD when the area under the curve (AUC) is@ 0.6 and >0.6. *p* < 0.05 indicates a statistical difference.

### 2.4. Identification of Ferroptosis Modification Pattern

According to the expression of DE-FRGs, unsupervised cluster analysis was performed to divide DKD samples into diverse ferroptosis modification patterns. A consistent clustering algorithm evaluated the clustering number and robustness. The R package “ConsensuClusterPlus” implements the above steps for 1000 iterations to guarantee the robustness of classification. Besides, the cumulative distribution function (CDF) curve of the consensus integral determines the optimal cluster number, the explicit separation of the consensus matrix heat map, the characteristics of the cumulative distribution function diagram, and the reasonable consensus values among cluster members. Principal component analysis (PCA) was used to verify the hub DE-FRG clustering effect.

### 2.5. GSVA and GO and KEGG Enrichment Analyses

We used the “GSVA” package to assess enrichment on the heat map. Downloaded the “c2.cp. kegg.v7.2.symbols.gmt” from the MSigDB database for analysis. Data were adjusted using the “limma” software package, and *p* < 0.05 indicated a significant difference among the subgroups. An adjusted *p* value < 0.05 among the subgroups was considered statistically significant. Biological signaling pathways are good at reflecting on biological changes; we use GO and KEGG enrichment analyses to determine underlying molecular mechanisms of DEGs between different ferroptosis modification patterns.

### 2.6. Landscape of Immune Infiltration and Immune Checkpoint Genes between Ferroptosis Modification Patterns

The proportions of twenty-three infiltrating immune cell types were quantified using “GSVA” in R package based on the ssGSEA strategy. In simple terms, ssGSEA calculates the individual enrichment score (ES) for each pair of samples and gene set using GSVA and GSEA base R packages [26]. Each GSEA ES represents the degree to which genes in a particular gene concentration are synergistically upregulated or downregulated in the sample. CIBERSORT deconvolution algorithm can be calculated according to the RNA matrix in proportion to the body's immune cells (https://cibersort.stanford.edu/) [27]. The abundance of immune cell infiltration and HLA gene expression among the distinct modification patterns was compared by the Wilcox test. More importantly, we also analyzed differences in immune checkpoint gene expression. The Spearman correlation analysis was used to analyze the relationship between hub DE-FRGs and immune cell infiltration.

### 2.7. Establishment of a Ferroptosis Score Signature

Hub DE-FRGs with positive and negative correlation with the clusters were named the ferroptosis gene features A and B, respectively. The Boruta algorithm can achieve dimensionality reduction of the ferroptosis gene features A and B. Besides, principal component analysis (PCA) was performed to extract principal component 1 as the characteristic fraction. The ferroptosis score index for each sample was then calculated using a method similar to the gene expression rating as follows: ferroptosis score = ∑PC1B − ∑PC1A, where PC1A represents the first component of feature A, PC1B represents the first component of feature B, and the scores of ferroptosis modification patterns and genotyping were detected [28]. Use R software “ggalluvial” package to draw Sankey.

### 2.8. Core Target Docking and Traditional Chinese Medicine (TCM) Prediction

The hub DE-FRGs, GO enrichment, and immune-related BP of the two groups was imported into the Coremine Medical database (http://www.coremine.com/medical/), and drugs with potential immunomodulator effects were screened [29]. Through the traditional Chinese medicine system pharmacology database and analysis platform (TCMSP) (http://lsp.nwu.edu.cn/tcmsp.php), the potential chemical components of traditional Chinese medicine were screened, and the oral bioavailability (OB) was set to be ≥30% and similar to the drug sex (DL) ≥ 0.20 as the threshold. Then, the effective targets were deduplicated and normalized using the UniProt prediction database. The processed data were imported into the Cytoscape 3.7.1 software to construct a “traditional Chinese medicine-active ingredient-target-channel-disease” network diagram.

### 2.9. Active Compound-Target Docking

Target proteins were downloaded from the TCMSP (https://old.tcmsp-e.com/tcmsp.php), the PDB database (https://www.rcsb.org/), and UniProt data (https://www.uniprot.org/). Models with less than 2.5 Å ligands binding were selected, and crystal structure was then entered into the PyMOL software (https://pymol.org/2/) for ligand dehydration, hydrogenation, and separation. AutoDock Vina 1.5.6 was then used to build docking grid boxes of crystal structures for each target. The molecule with the lowest binding energy in the docking configuration was selected for visualization and compared with the interaction between protein and small molecule ligand to observe its binding effect, such as hydrophobicity, cation-*π*, anion-*π*, *π*-*π* stacking, and hydrogen bonding. According to the docking score of AutoDock Vina, Affinity represents the strength of the binding force, Affinity > −4 kcal·mol, and the binding force is extremely weak or considered as no binding force. −7 kcal · mol < Affinity < −4 kcal · mol is defined as medium binding force; Affinity < −7 kcal · mol is defined as a standard with solid binding force [30].

### 2.10. Statistical Analyses

The R software (version 4.0.5) was used for the analyses. Comparisons of two groups to determine significance was done by the Wilcoxon test, and the significance among more than three groups was identified using the Kruskal-Wallis test. Evaluation of the models' predictive accuracy was done using ROC curves and the corresponding area under ROC curve (AUC) values. Spearman's correlation analysis was employed for description of correlations between quantitative variables that were not normally distributed. Two-tailed *p* ≤ 0.05 was the threshold for significance.

## 3. Results

### 3.1. Identification of DE-FRGs

**Figure 2:**
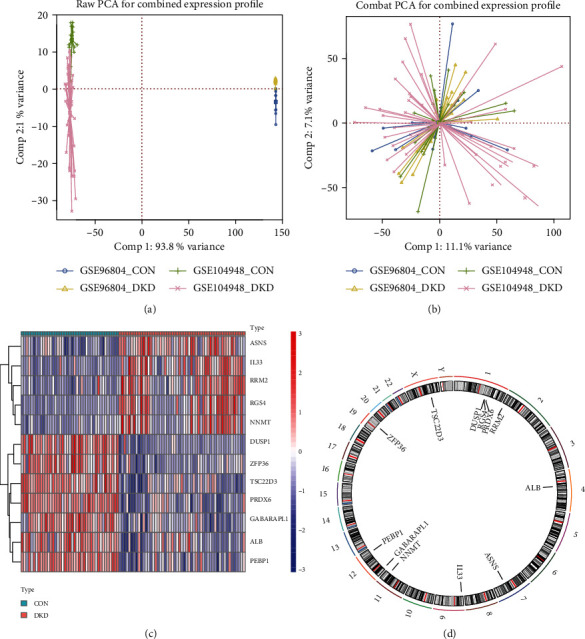
Data preprocessing. (a) PCA was used to remove batch effect. (b) Before batch correction and after batch correction. (c) Heat map showed the transcriptome expression of DE-FRGs between DKD and CON samples. (d) Chromosomal distribution of DE-FRGs in DKD.

PCA clustering was employed to calculate and form overlapping or predefined clusters approximately. Before the batch adjustment, the microarray data sets were validated on multiple platforms. Following the batch effect, samples from every dataset amalgamate into each other. The batch effect was significant before the combination of datasets GSE96804 and GSE104948 (Figure 2(a); Comp 1: 78.6% variance, Comp 2: 5.7% variance), after batch calibration (Figure 2(b); Comp 1: 78.6% variance, Comp 2: 5.7% variance). The combined dataset contains 53 DKD and 41 control (CON) samples, extracting 138 ferroptosis genes. DE-FRGs were analyzed using the |log2FC| ≥ 1 and adjusted *p* < 0.05 DE-FRGs. 12 DE-FRGs were obtained, including five upregulated genes and seven downregulated genes (Figure 2(c)). Chromosomal localization showed that DE-FRGs were mainly located on chromosomes 1 and 12. Chromosome 1 contains one suppressor and two mark ferroptosis genes. Ferroptosis driver genes are located on chromosome 12 Figure 2(d).

### 3.2. Screening for Hub DE-FRGs

**Figure 3:**
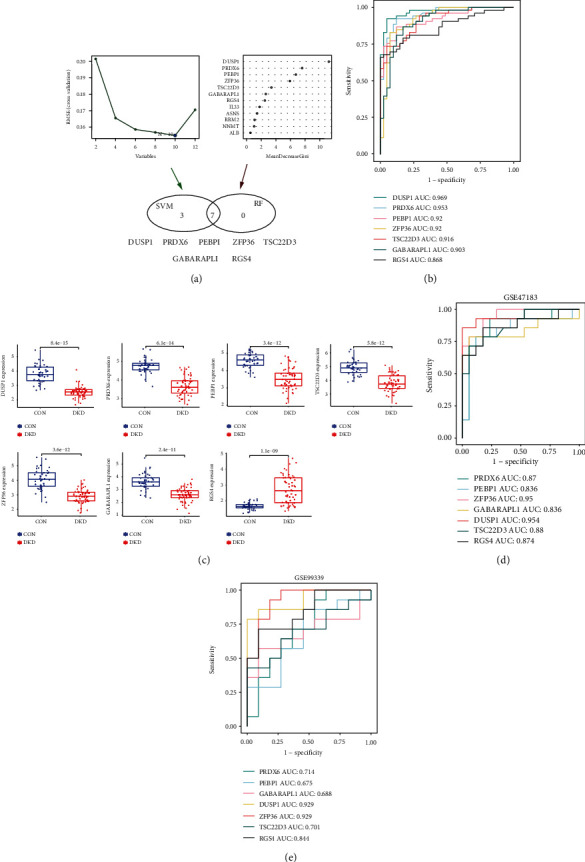
Screening for hub DE-FRGs. (a) DKD disease diagnosis genes were selected by RF and SVM. (b) Subject operating characteristic curve analysis of hub DE-FRGs in internal set classification model. AUC value and area under ROC curve. (c) The box plot showed the difference of hub DE-FRG expression between DKD and CON samples. (d, e) The area under the curve of AUC values of GSE47183 and GSE99339.

We apply the representation data of 12 DE-FRGs to the machine learning algorithm of the RF classifier. All genes were ordered by “average decreased accuracy” and “average decreased Gini coefficient.” The larger the two values are, the closer the relationship between DE-FRGs and DKD. RF screening results showed that there were 7 DE-FRGs with an importance score > 2, including DUSP1, PRDX6, ZFP36, TSC22D3, PEBP1, RGS4, and GABARAPL1. Using the SVM algorithm, we obtained ten feature signatures when the root mean square error (RMSE) is minimum, including DUSP1, PRDX6, ZFP36, PEBP1, TSC22D3, GABARAPL1, RGS4, IL33, RRM2, and ASNS. The blue dots indicate the number of signatures with the highest accuracy or minimum error. The intersection genes of the two machine algorithms were selected as hub DE-FRGs, and seven hub DE-FRGs were obtained, including Dual specificity phosphatase 1 (DUSP1), peroxidase 6 (PRDX6), phosphatidylethanolamine-binding protein 1 (PEBP1), ZFP36 ring finger protein (ZFP36), glucocorticoid-induced leucine zipper (TSC22D3), GABA type A receptor-associated protein-like 1 (GABARAPL1), and regulator of G protein signaling 4 (RGS4) (Figure 3(a)). To identify the diagnostic efficacy of DUSP1, PRDX6, PEBP1, ZFP36, TSC22D3, GABARAPL1, and RGS4 distinguishing between DKD and CON, ROC analysis was performed by utilizing the data of the internal set. The AUC was 0.969 for DUSP1, 0.953 for PRDX6, 0.92 for PEBP1, 0.92 for ZFP36, 0.916 for TSC22D3, 0.903 for GABARAPL1,and 0.868 for RGS4 (Figure 3(b)). Then, the Wilcoxon test was used to compare the expression differences of 7 hub DE-FRGs in DKD samples and CON samples, and the results showed that DUSP1, PRDX6, PEBP1, ZFP36, TSC22D3, and GABARAPL1 were significantly reduced in DKD samples (*p* < 0.05). RGS4 was significantly up in DKD samples (*p* < 0.05) (Figure 3(c)). DUSP1, TSC22D3, and RGS4 are mark-ferroptosis genes; ZFP36 and PRDX6 are suppressor-ferroptosis genes; PEBP1 and GABARAPL1 are driver-ferroptosis genes. In the independent external set (GSE47183), the AUC of 7 hubs DE-FRGs was more significant than 0.8 (Figure 3(d)). In the independent external set (GSE99339), the AUC of 7 hubs DE-FRGs was more significant than 0.6 (Figure 3(e)). The box plot showed that the expression of 7 hub DE-FRGs in DKD and CON samples was consistent with the internal set (Figure S1(a, b)). Thus, seven hub DE-FRGs might be a potential diagnostic biomarker of DKD.

### 3.3. Ferroptosis Modification Patterns Mediated by Hub DE-FRGs in DKD

**Figure 4:**
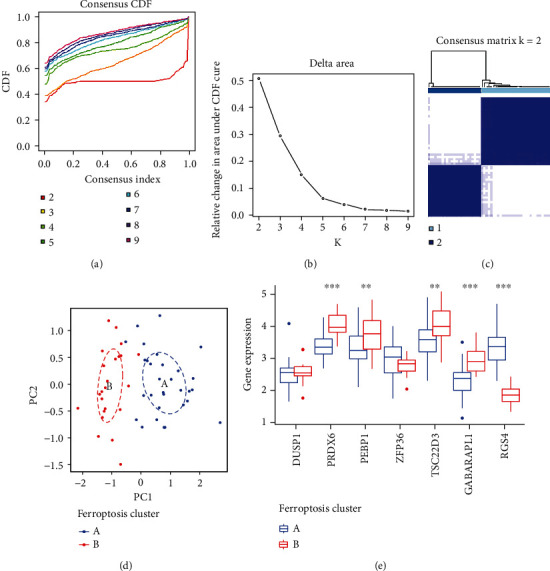
Unsupervised clustering of hub DE-FRG regulators identified two different ferroptosis modification patterns in DKD. (a) Cumulative distribution function (CDF) is displayed for *k* = 2–9. (b) The relative change in area under the CDF curve for *k* = 2–9. (c) The correlation between subgroups when cluster numbers *k* = 2. (d) PCA analysis of transcriptome profiles of two ferroptosis modification patterns showed significant differences among different modification modes. (e) The expression difference of 7 hub DE-FRGs in the two ferroptosis patterns. ^∗^*p* < 0.05, ^∗∗^*p* < 0.01, and ^∗∗∗^*p* < 0.001.

To investigate ferroptosis modification patterns in DKD, we performed an unsupervised consensus clustering analysis of DKD samples based on the expression hub DE-FRG (DUSP1, PRDX6, PEBP1, ZFP36, GABARAP1, TSC22D3, and RGS4) (Figures 4(a)–4(c)). We identified two different ferroptosis modification patterns, and the expression of seven hub DE-FRGs was qualitatively different, including 30 DKD samples in pattern A and 23 DKD samples in pattern B (Figure 4(d)). Five hub ferroptosis-DEGs have remarkable differences in expression between 2 ferroptosis modification patterns (*p* < 0.05). PRDX6, PEBP1, TSC22D3, and GABARAPL1 were significantly upregulated in pattern B. RGS4 was significantly downregulated in pattern A (Figure 4(e)), validating the existence of diverse ferroptosis modification patterns in DKD.

### 3.4. Biological Characteristics of Ferroptosis Modification Patterns

**Figure 5:**
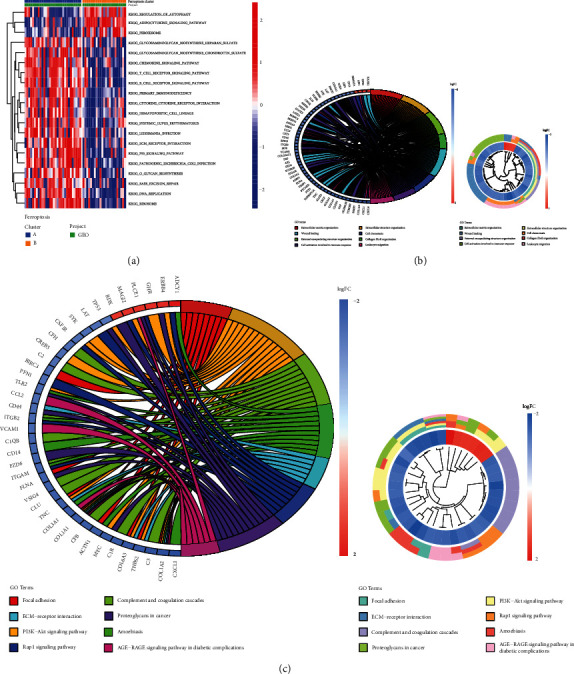
Enrichment analysis. (a) The heat map was used to visualize the biological processes assessed by GSVA. (b) Function annotation DEGs using GO terms and (c) KEGG pathway.

GSVA enrichment was performed to explore the two clusters' biological behavior and pathway differences. Pattern B was significantly enriched in the regulation of autophagy and peroxisome pathways, and pattern A showed enrichment in immune-inflammatory pathways (Figure 5(a)). Examples include the T cell receptor signaling pathway and chemokine signaling pathway, B cell receptor signaling pathway, P53 signaling pathway, and primary immunodeficiency. In order to further interpret the clustering results from the perspective of fundamental biological processes, we performed GO and KEGG analyses on genes that are differentially expressed between pattern A and pattern B. A total of 709 DGEs were obtained. According to the results of the GO analysis, DGEs were primarily enriched in cell activation involved in immune response, leukocyte migration, and T cell activation (Figure 5(b)). The results of the KEGG analysis showed that these DEGs were significantly enriched in PI3K-Akt signaling pathway, complement, coagulation cascades, ECM-receptor interaction, Rap1 signaling pathway, proteoglycans in cancer, and AGE-RAGE signaling pathway in diabetic complications (Figure 5(c)). These results suggest that DEGs between patterns A and B may be involved in the inflammatory immune response of DKD.

### 3.5. Immune Microenvironment Characteristics in Distinct Ferroptosis Modification Patterns

**Figure 6:**
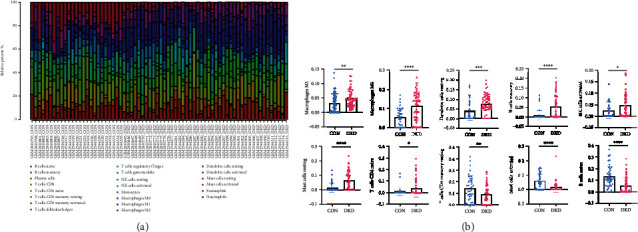
Analysis of immune cell infiltration. (a) The bar plot showing the proportion of infiltrated immune cells calculated by the CIBERSORT algorithm. (b) Box plots showing the abundance of differentially expressed immune cells in CON and DKD. ^∗^*p* < 0.05, ^∗∗^*p* < 0.01, and ^∗∗∗^*p* < 0.001.

**Figure 7:**
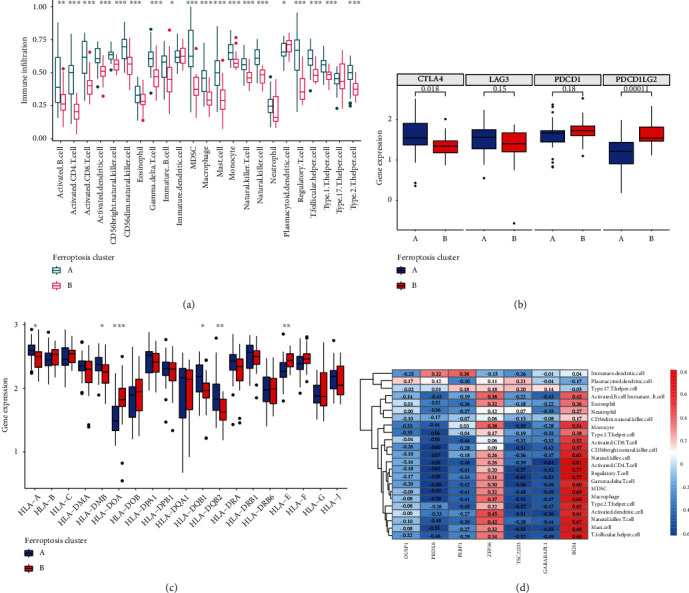
Immune cell landscape in different ferroptosis modification patterns in DKD. (a) Box plots show the differences in immune cell infiltration calculated by ssGSEA algorithm in different modes of iron death modification. (b) Differential expression of immune checkpoint genes between different ferroptosis modification patterns. (c) Box plot of differential expression of HLA-related genes. (d) Heat map of the correlation between core genes and immune cells. ^∗^*p* < 0.05, ^∗∗^*p* < 0.01, and ^∗∗∗^*p* < 0.001.

Based on the CIBERSORT deconvolution algorithm, we found a significant difference in the proportion of immune cells between DKD and CON (Figure 6(a)). Box plot also shows that macrophages M1 and M2, B cell memory, T cell CD4 naive, NK cell activated, dendritic cell resting, and mast cell resting were significantly increased in DKD samples, while B cell naive, T cell CD4 memory resting, and mast cell activated were significantly decreased (*p* < 0.05) (Figure 6(b)). Furthermore, using the ssGSEA algorithm to quantify the abundance of immune cell infiltration in the two ferroptosis modification patterns, we found that many immune cell infiltration degrees differed between the two patterns. This conclusion is consistent with the previous GSVA enrichment results. We found that the majority of the infiltrated immune cells were rich in pattern A; however, there was no statistically significant difference in eosinophil, immature dendritic cell, neutrophil, and type 17 T helper cell between the two patterns (Figure 7(a)). Only four immune checkpoints (CTLA4, LAG3, PDCD1, and PDCD1LG2) were retrieved in the combined validation set, in which CTLA4 was significantly downregulated in pattern B and PDCD1LG2 was significantly upregulated (*p* > 0.05) (Figure 7(b)). HLA gene expression trends showed that HLA-A, HLA-DMB, HLA-DQB1, and HLA-DQB2 in pattern A were significantly higher expressions than those in pattern A (*p* < 0.05) (Figure 7(b)). The correlation heat map shows that PRDX6 is negatively correlated with immune cells, while RGS4 is positively correlated with immune cells (*p* < 0.05) (Figure 7(c)). According to the expression level of PRDX6, DKD samples were divided into the PRDX6 high expression group and PRDX6 low expression group, and the abundance of immune cell infiltration was detected. The results showed that the PRDX6 high expression group had significantly decreased immune cells except for immature B cell, immature dendritic cell, neutrophil, plasmacytoid dendritic cell, type 17 T helper cell, and type 2 T helper cell (*p* < 0.05) (Figure S2(a)). In addition to eosinophil, immature dendritic cell, neutrophil, plasmacytoid dendritic cell, and type 17 T helper cell, the abundance of immune cells in the high expression group of RGS4 was significantly increased (*p* < 0.05) (Figure S2(b)). The differences in immune cell infiltration were consistent with the distinct ferroptosis modification patterns. High expression of pattern B DE-FRGs (PRDX6, PEBP1, GABARAPL1, and TSC22D3) and immune cells and HLA genes was negatively correlated. The above results proved that ferroptosis modification had a negative regulatory role in shaping different immune microenvironments in DKD.

### 3.6. Identification of Ferroptosis-Related Gene Clusters and Ferroptosis Score in DKD

**Figure 8:**
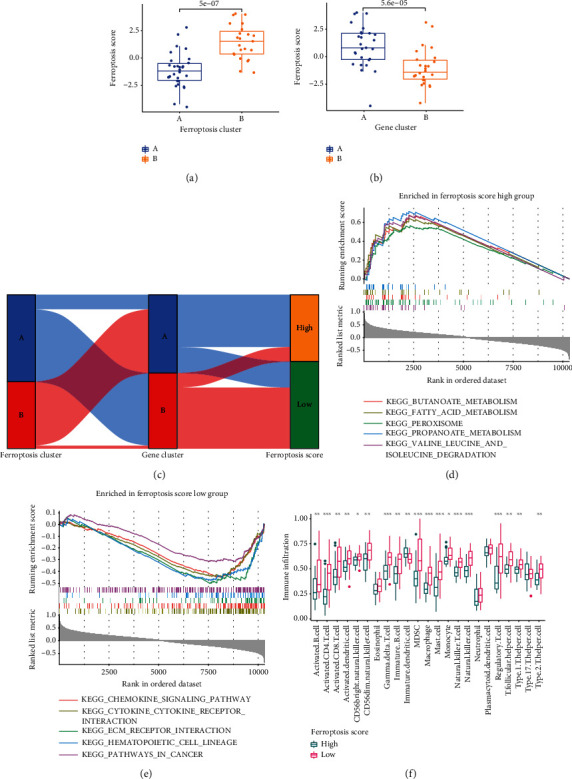
Ferroptosis scores in different clusters. (a) Ferroptosis score box plot in ferroptosis modification patterns. (b) Ferroptosis score box plot in gene clusters. (c) Alluvial diagram of ferroptosis score with different ferroptosis modification patterns and gene clustering. (d, e) GSEA enrichment analysis of high and low ferroptosis score groups. (f) High and low ferroptosis score group immune cell infiltration. ^∗^*p* < 0.05, ^∗∗^*p* < 0.01, and ^∗∗∗^*p* < 0.001.

We obtained a total of 709 DEGs related to patterns A and B. Based on these DEGs, we repeated the unsupervised consistency cluster analysis to obtain the gene clusters A and B, with 27 DKD samples in cluster A and 26 DKD samples in cluster B (Figure S3 (a-c)). The box plot showed that hub DE-FRGs were expressed between clusters A and B (Figure S3(d)). Three hub DE-FRGs (PRDX6, GABARAPL1, and TSC22D3) were significantly upregulated in cluster A compared with cluster B. ZFP36 and RGS4 were significantly upregulated in cluster B. GSVA enrichment revealed that gene cluster A was mainly enriched in inositol phosphate metabolism and regulation of autophagy biological functions, while gene cluster B was related to the biological functions or pathways such as glycan biosynthesis, glycosaminoglycan biosynthesis, chondroitin sulfate, P53 signaling pathway, and primary immunodeficiency (Figure S3(e)). Analysis of ssGSEA immune cell infiltration showed that immune cell infiltration was significantly higher in gene cluster B than in cluster A, except for plasmacytoid dendritic cells (Figure S3(f)). The box plot showed that CTLA4 significantly decreased in cluster A, while PDCD1LG2 significantly increased (*p* > 0.05) (Figure S3(g)). HLA-DMB, HLA-DQB1, and HLA-DQB2 were significantly decreased in cluster A (*p* > 0.05), and the conclusion was similar to the ferroptosis modification pattern (Figure S3(h)). These results once again confirmed that iron death modification plays an important role in immune regulation of DKD. We calculated the ferroptosis scores of each patient with DKD using the equation ∑PC1B − ∑PC1A. According to the ferroptosis scores, the DKD samples were divided into high score groups and low score groups, as ferroptosis scores were significantly lower in pattern A than in pattern B (*p* < 0.05) (Figure 8(a)). The ferroptosis scores of cluster A in the gene cluster were significantly higher than that of cluster B (*p* < 0.05) (Figure 8(b)). The ferroptosis scores of ferroptosis modification pattern and gene cluster were displayed in an alluvial diagram based on the “ggalluvial” package (Figure 8(c)). These partial results showed high similarity between ferroptosis modification pattern classification and gene cluster. Ferroptosis modification pattern A and gene cluster B mainly belong to low ferroptosis scores, while pattern B and cluster A mainly belong to high ferroptosis scores. GSEA revealed that high ferroptosis scores were enriched in ferroptosis-related pathways such as the fatty acid metabolism and peroxisome signaling pathways (Figure 8(d)). Low ferroptosis scores were enriched in the chemokine signaling pathway and ECM-receptor interaction (Figure 8(e)). Immune cell infiltration was decreased in high ferroptosis scores, except for immature dendritic cell (Figure 8(f)). This conclusion is consistent with the pattern of ferroptosis modification and the immune cell infiltration of gene clustering. These results indicate that ferroptosis scores are effective predictors of immune infiltration abundance in DKD.

### 3.7. Prediction of DKD-Related Potential Core Targets and Immune-Related Biological Processes in TCM

**Figure 9:**
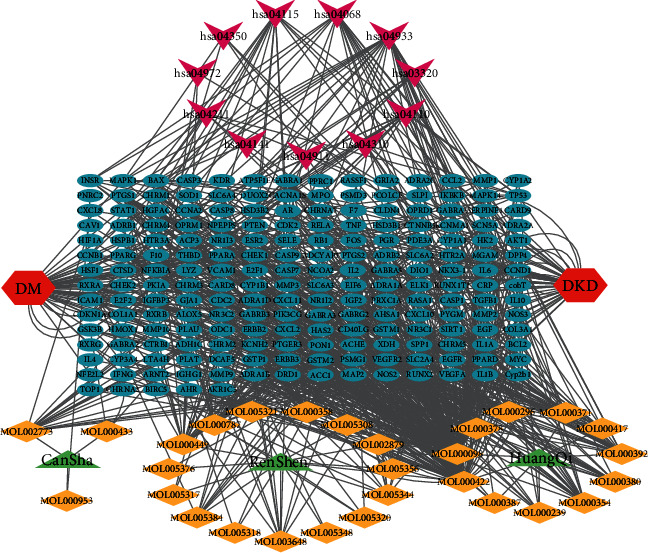
“TCM-active component-target-pathway-disease” action network.

The biological processes related to GO and KEGG immunity screened out by hub DE-FRGs were imported into the Coremine Medical database. The threshold condition was set as *p* < 0.05 to screen out the traditional Chinese medicines related to hub DE-FRGs and immune-related biological processes. The predicted results showed the relationship between hub DE-FRGs (DUSP1, PRDX6, PEBP1, ZFP36, GABARAPL1, and RGS4) as well as biological processes such as T cell receptor signaling pathway, natural killer cell-mediated cytotoxicity, macrophage differentiation, T cell activation, and T cell differentiation. A total of 52 Chinese medicines were found to be closely related to immunity and hub DE-FRGs (Table S1). TSC22D3 and immune-related TCM were not retrieved. The results showed that Bombyx mori L (Can Sha), Panax ginseng C A Mey (Ren Shen), and Hedysarum multijugum Maxim (Huang Qi) had the highest repeatability. Using the TCMSP database, compounds were screened with oral bioavailability (OB) ≥ 30% and drug similarity (DL) ≥ 0.18 as thresholds. The TCMSP database predicted compound targets, and the effective targets were deweighted and normalized by the UniProt database. A total of 201 potential targets of drug compounds were predicted by using TCMSP database, of which 17 targets were involved in the “FoxO signaling pathway” related to diabetic nephropathy. Seven targets participated in “longevity regulating pathway”; 26 targets were involved in “AGE-RAGE signaling pathway in diabetic complications”; 6 targets were involved in type 2 DM-related signaling pathway “PPAR signaling pathway”; 13 targets participated in “cell cycle”; 14 targets were involved in the p53 signaling pathway; 6 targets involved in Wnt signaling pathway. Use Cytoscape to construct the “TCM-active component-target-pathway-diseases” action network (Figure 9). The green triangle represents the medicine, the yellow diamond represents the active ingredient, the blue circles represent compound targets, the red hexagon represents disease, and the magenta triangle represents disease-related signaling pathways. Thus, the top two degree values are that active ingredient kaempferol (MOL000422) of Ren Shen and quercetin (MOL000098) of Huang Qi, with each other through acting on multiple different targets to realize network regulation effect, thus causing complex regulatory relationship and thus exerting a therapeutic effect. In addition, kaempferol is both the active ingredient of Ren Shen and Huang Qi and ranks top two in the medium value of Cytoscape. It is suggested that the active ingredient may be a potential candidate drug for immunotherapy of DKD.

### 3.8. Compound-Target Docking

**Figure 10:**
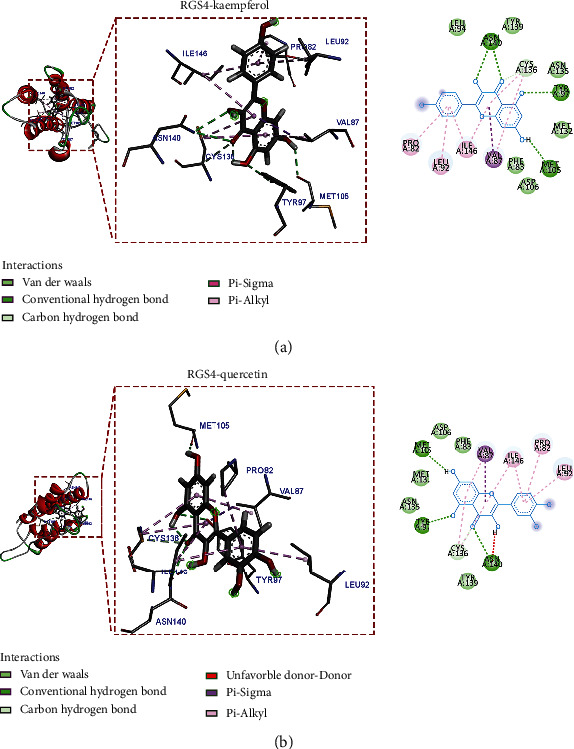
Molecular docking model of the core active ingredients with RGS4 (PDB: 5CFW). (a) The molecular docking of kaempferol with RGS4. (b) The molecular docking of quercetin with RGS4.

**Figure 11:**
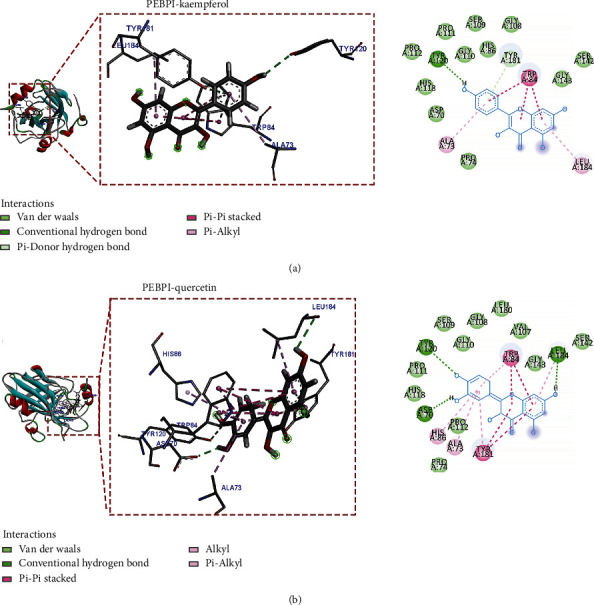
Molecular docking model of the core active ingredients with PEBP1 (PDB: 2QYQ). (a) The molecular docking of kaempferol with PEBP1. (b) The molecular docking of quercetin with PEBP1.

Compounds kaempferol and quercetin, which ranked the top two in the degree value of a drug-component-target network, were selected for molecular docking of disease core targets. The Chem3D software was employed to draw the corresponding 3D structure according to the structural formulas of the two practical active components and output in mol^∗^2 format. The 3D structures of core proteins DUSP1 (PDB: 6APX), PRDX6 (PDB: 1PRX), PEBP1 (PDB: 2QYQ), GABARAPL1 (PDB: 2R2Q), and RGS4 (PDB: 5CFW) were downloaded from the PDB database and output in PDB format. We did not retrieve ZFP36 and TSC22D3 proteins that met the inclusion criteria in the PDB and UniProt databases. The AutoDockTools 1.5.6 software was used to convert active components and core proteins into PDBQT format to find functional pockets; that is, the ligand is combined with one or more amino acid residues to form H bonds, *P-π* bonds, or *π-π* bonds, and other active sites and calculate the binding energy. The binding energies of the main active ingredients and the protein encoded by target genes are all less than -6.0 kcal/mol (Table 2), indicating good binding activity. The results of AutoDock Vina docking showed that kaempferol, the main active component of Ren Shen, formed the most stable molecular docking with RGS4, and the docking binding energy was -8.5 kcal/mol. Quercetin, the main active component of Huang Qi, formed the most stable molecular docking with PEBP1. The docking combined energy is -8.6 kcal/mol. Kaempferol forms four hydrogen bonds with TYR97, ASN140, and MET105 of amino acid residues of RGS4; CYS136 forms a C-H bond with the ligand; PRO82, LEU92, ILE146, VAL87, and CYS136 formed *p-π* conjugation effect with ligand. Moreover, a *p-π* hydrophobic stacking interaction is formed between VAL87 and the ligand. In addition, hydrophobic amino acids surround, such as LEU94, TYR139, ASN135, MET132, ASP106, and PHE83 (Figure 10(a)). Quercetin forms three hydrogen bonds with amino acid residues MET105, TYR97, and ASN140 of RGS4 (PDB: 5CFW). ILE146, PRO82, LEU92, and CYS136 formed a *p-π* conjugation effect with ligands; VAL87 formed a *p-π* hydrophobic interaction with its ligand. There are also hydrophobic amino acids nearby (Figure 10(b)). It is speculated that ASN140, CYS136, and VAL87 play critical roles in the interaction between RGS4 and the ligand. Kaempferol forms a hydrogen bond with amino acid residue TYR120 of PEBP1 and a C-H bond with TYR181; TRP84 and ligand form stable *π*-*π* hydrophobic stacking; ALA73 and LEU184 form p-*π* conjugation effects with ligands. Moreover, there is a hydrophobic interaction with many hydrophobic amino acids (Figure 11(a)). Quercetin forms three hydrogen bonds with amino acid residues TYR120, LEU184, and ASP70 of PEBP1; TRP84 and TYR181 formed stable *π*-*π* hydrophobic stacking and *p-π* conjugation with ligands; LEU184, HIS86, and ALA73 form *p-π* conjugacy effects with ligands (Figure 11(b)). It is speculated that TRP84 and TYR120 play a vital role in the interaction between PEBP1 and the ligand.

In the crystal structure of the receptor protein molecule DUSP1 (PDB: 6APX), through a series of simulation calculations, ARG492 and HIS388 may be the active site residues of the receptor (Figure S4 (a, b)). The crystal structure of PRDX6 (PDB: 1PRX) suggests that LYS67 and LYS199 may be the active site residues of the receptor (Figure S5 (a, b)). LEU50 and ARG67 may be active site residues of the GABARAPL1 (PDB: 2R2Q) receptor (Figure S6 (a, b)). Our data suggested the direct binding of Chinese herbal compounds kaempferol and quercetin to its target proteins DUSP1, PRDX6, PEBP1, GABARAPL1, and RGS4.

## 4. Discussion

In order to reveal how ferroptosis affects the immune response of DKD patients and the effect of activation of immune pathways on immune cells, this study constructed different ferroptosis abundance clusters. It evaluated the correlation between different ferroptosis abundance clusters and immune infiltration. We found that the expression of most ferroptosis regulators was altered after the development of DKD. The ferroptosis-related genes (DUSP1, PRDX6, PEBP1, ZFP36, GABARAPL1, TSC22D3, and RGS4) were closely related to DKD disease by the random forest tree and support vector machine model we constructed. Ferroptosis marker genes (DUSP1 and TSC22D3), ferroptosis suppressor genes (ZFP36 and PRDX6), and ferroptosis driver genes (PEBP1 and GABARAPL1) were significantly decreased, while ferroptosis marker gene RGS4 was significantly increased. When evaluating the model screening results, it was found that the differential ferroptosis genes obtained by the machine algorithm model can distinguish DKD from regular patients and have better diagnostic and distinguishing value, which is conducive to developing clinical-specific antibodies. These genes have also been reported in some studies. Animal experiments have shown that under high glucose (HG) environment, PRDX6 in mouse podocyte MPC5 is significantly reduced. PRDX6 overexpression can increase podocyte viability and inhibit high glucose-induced ROS and MDA production. It increases the expression of SLC7A11 and GPX4 and inhibits ferroptosis. Interestingly, PRDX6 reduced the activation of plasma NF-*κ*B and TNF-*α* levels in diabetic mice [31]. These results strongly suggest that PRDX6 can improve renal function by inhibiting ferroptosis, which may be closely related to improving inflammatory response. This conclusion is consistent with the results of machine algorithm analysis. The decrease of PRDX6 transcriptome level in DKD will aggravate the process of ferroptosis and inflammatory response.

Another molecule derived from model evaluation, PEBP1, also known as RAF-1 kinase inhibitory protein (RKIP), is a member of the phosphatidylethanolamine-binding protein family [32]. It is well known that NF-*κ*B signaling is essential in regulating oxidative stress and inflammation. Downregulating, the expression of its pathway proteins can inhibit the upregulation of inflammatory cytokines [33]. Studies have found that RKIP can act as a negative regulator of the nuclear factor kappa B (NF-*κ*B) signaling pathway, inhibit its expression, and slow the inflammatory response, which undoubtedly has a protective regulatory effect on DKD [34]. Similarly, ZFP36 obtained from model screening is a zinc finger protein affecting TNF-*α* mRNA's stability. Zhang et al. found that the inactivation of ZFP36 in mice resulted in a complex inflammatory syndrome caused by increased TNF-*α* production [35]. Some studies have reported that the level of ZFP36 in diabetic patients was significantly lower than in controls. In addition, the levels of IL-6 and IL-18 in serum and urine of patients with proteinuria were significantly increased, while the level of ZFP36 was significantly lower than that of patients without proteinuria or microproteinuria. Serum ZFP36 was negatively correlated with IL-6/IL-18 [36]. Iron overload aggravates renal cell damage and is closely related to inflammation and immune regulation. The above evidence undoubtedly confirms that ferroptosis affects renal function in DKD patients and is related to inflammation. Processes such as metabolism, hypoxia, and apoptosis are also involved in disease regulation [37]. For example, the low expression of ferroptosis-related gene DUSP1 obtained by screening is related to glucose metabolism disorder, renal failure, renal hypertrophy, renal fibrosis, and glomerular apoptosis. At the molecular level, defective DUSP1 expression activates the JNK pathway, which selectively initiates mitochondrial fission by regulating mitochondrial fission factor (Mff) phosphorylation. DUSP1 overexpression suppresses glomerular apoptosis and prevents mitochondrial damage induced by high glucose stress. Mechanically, reintroduction of DUSP1 inactivates the JNK pathway and alleviates Mff phosphorylation. Inactive Mff disrupts hyperglycemia-mediated mitochondrial fission and thus reduces mitochondrial oxidative stress, represses mPTP opening, weakens proapoptotic leakage into the cytoplasm, and closes mitochondria-dependent cellular apoptosis in the setting of diabetes [38]. In addition, as glucose metabolism disorders cause chronic microvascular lesions, insulin secretion can directly or indirectly affect the progression of DKD. In this study, RGS4 was found to be significantly changed in the progression of DKD patients. RGS4 is a vascular-associated RGS protein that functions as a GTPase activator of Gq and Gi family members. RGS4 also helps reduce angiotensin-dependent superoxide production, prevents vascular oxidation, and may be involved in inhibiting cell signaling through CXC motif chemokine receptors [39]. According to Bastin et al., in phases I and II of insulin release from intact mice and isolated islets, loss of RGS4 resulted in A marked deficiency of glucose-stimulated insulin secretion [40]. In addition, the research group previously found that hypoxia regulation can be involved in the regulation process of DKD. In the results of this study, TSC22D3, also known as glucocorticoid-induced leucine zipper, is a glucocorticoid other than DUSP1. One of the reactive anti-inflammatory molecules regulates intracellular signaling pathways through HIF-1*α* and AP-1 [41, 42]. TSC22D3 inhibits hypoxia- and diabetes-induced galectin-1 expression due to hypoxia and diabetes by disrupting the stability of the HIF-1*α* protein. In addition, GABARAPL1, also known as autophagy-associated protein 8 (ATG8), is a ubiquitin-like protein involved in autophagosome formation. In our study, we found that the expression of this gene was downregulated in DKD samples. Zhang et al. demonstrated that GABARAPL1 acts as a tumor suppressor and inhibits Wnt signaling by mediating Dvl2 degradation through the autophagy pathway [43]. On the other hand, DM has been reported to induce Wnt1/*β*-catenin signaling, promote damage to podocytes, induce epithelial-mesenchymal transition, and worsen kidney injury and fibrosis [44]. However, whether GABARAPL1 can improve the progress of DKD through this pathway needs further exploration.

In our study, unsupervised consistent clustering analysis was performed in DKD patients based on hub DE-FRG expression to obtain two distinct patterns of ferroptosis modification. Then GSVA was used to analyze the signal pathway enrichment of the two ferroptosis clustering patterns, and the differential genes between the two patterns were screened for GO and KEGG enrichment analysis. The results showed that the hub DE-FRG high expression group (pattern B) was significantly enriched in autophagy and peroxidase pathways, while pattern A was significantly enriched in immune-related pathways. Growing evidence suggests that T lymphocyte activation and cytokine-induced inflammation are closely related to type DM and DKD [45].CTLA4 interacts with CD28 on T cells, which is essential for T cell activation. Previous work suggested that treatment with CTLA4-Ig (abatacept), a molecule that binds and blocks B7-1 and is licensed for the treatment of rheumatoid arthritis, could ameliorate DKD. Animal studies have also shown that CTLA4 enhances autoimmune responses in DM mice [46, 47]. NF-*κ*B signaling pathway is closely related to apoptosis and participates in the transcriptional regulation of various apoptosis-related genes, which is an important way to regulate immunity. There are four major genes in this pathway, including programmed cell death 1 (PDCD1) and PDCD1LG2. The abnormal expression of Fas cell surface death receptor (FAS) and Fas ligand FASLG has been shown to be associated with some autoimmune diseases [48, 49]. However, our results showed that CTLA4 was significantly decreased in the high expression group of hub DE-FRGs (pattern B), while PDCD1LG2 was significantly increased. Differential analysis of HLA genes also showed that HLA-A, HLA-DMB, HLA-DQB1, and HLA-DQB2 were significantly higher in pattern A than in pattern B. HLA-DQB1 and HLA-DQB2 belonged to HLA class II molecules mainly distributed on the surface of B cells, monocyte-macrophage, and dendritic cells, involved in the presentation and immune regulation of exogenous antigens [50]. GO and KEGG enrichment analyses also showed that the differential genes between pattern A and pattern B were mainly enriched in the immune and glucose metabolism-related pathways. These results suggest that ferroptosis plays an essential role in the immune microenvironment of DKD. To validate the accuracy of ferroptosis clustering and the relationship between different modification patterns and immune microenvironment, we performed a second unsupervised DEG consensus cluster based on subcohort screening, resulting in two gene clusters. The ferroptosis score of each DKD patient was calculated, and the DKD patients were divided into high and low score groups. The relationship between gene clustering and ferroptosis score and immune microenvironment was compared. Gene clustering results were divided into cluster A and cluster B. Gene cluster B had low expression of ferroptosis genes, except for RGS4 and ZFP36. Immune cells were mainly enriched in cluster B, and HLA-DMA, HLA-DQB1, and HLA-DQB2 were significantly increased in cluster B. GSVA enrichment results show that cluster B is mainly enriched in glycan biosynthesis, glycosaminoglycan biosynthesis chondroitin sulfate, P53 signaling pathway, and primary immunodeficiency. The above conclusion is similar to ferroptosis modification pattern A. The infiltration of immune cells in the high ferroptosis score group was significantly lower than in the low ferroptosis score group. GSEA analysis showed that the high score groups were mainly enriched in ferroptosis-related signaling pathways, such as fatty acid metabolism and peroxisome signaling pathways. This result is similar to ferroptosis modification pattern B enrichment previously analyzed by GSVA enrichment. Based on the immune characteristics of the above subtypes, the reliability of our classification of DKD immunophenotypes was further confirmed. In addition, the ferroptosis score was negatively correlated with infiltrating immune cells in DKD patients. Ferroptosis score plays a role in predicting the effectiveness of immunotherapy in patients with DKD.

In this study, we found that the infiltration of macrophages, dendritic cells, B cells, NK cells, mast cells, CD4 T cells, and myeloid-derived suppressor cell (MDSC) immune cells in DKD glomeruli increased significantly but decreased significantly in ferroptosis modification pattern B and high ferroptosis score groups. Under high glucose stress, kidney cells produce proinflammatory responses that promote innate immune response and uptake of macrophages by releasing chemokines, cellular adhesion molecules, and damaging risk-associated molecular patterns (DAMPs). Its action activates and supplements cytokines, enhances innate immune response, and promotes infiltration of inflammatory cells and mast cells into kidney tissue [51]. As DKD progresses, the kidney becomes more sensitive to ischemia, and the infiltration of neutrophils and macrophages in renal tissue increases. Macrophages can secrete a variety of proinflammatory mediators, including interleukin- (IL-) 1*β*, tumor necrosis factor- (TNF-) *α*, monocyte chemokine, and chemotactic protein- (MCP-) 1, which in turn cause kidney inflammation and fibrosis, accelerating disease progression [52, 53]. Myeloid-derived suppressor cells (MDSC) are a subset of regulatory immune cells that inhibit other immune cells, including T, B, and NK [54]. There are two representative subtypes of MDSCs: polymorphonuclear MDSCs (PMN-MDscs) and monocyte MDSCs (M-MDscs). Islam et al. found that PMN-MDscs were significantly increased in DKD patients through clinical studies. The production of ROS in DKD PMN-MDSCs was higher than that of healthy neutrophils or immune cells and increased under hyperglycemic conditions [55]. These immune responses promote DKD and decreased renal function. Moreover, we found that PRDX6 had a negative regulatory relationship with immune cells, while RGS4 had a positive regulatory relationship with immune cells. Activated CD8 T cell, regulatory T cell, gamma delta T cell, mdsc, CD56 bright natural killer cell, natural killer cell, activated CD4 T cell, macrophage, and type 1 T helper cell (Th1) were negatively correlated with PRDX6 and positively correlated with RGS4. Moreover, RGS4 was positively correlated with type 2 T helper cell, activated dendritic cell, natural killer T cell, mast cell, and T follicular helper cell. CD8+T cells may increase the expression of IFN-*γ* and TNF-*α* through cytotoxic effects, thus promoting the progression of diabetic kidney [56]. Activated T cells have a high demand for iron, and iron deficiency inhibits T cell proliferation, whereas iron overload leads to an imbalance in the ratio of CD4 and CD8 T cells. IFN-*γ* released by CD8+T cells may activate the JAK1-STAT1 pathway to inhibit SLC7A11 expression, reducing cystine uptake by tumor cells and leading to iron death [57]. Alterations of Th1 cytokines in DM are associated with a worsening proinflammatory state and generation of oxidative stress [58]. Th1 cells can produce large amounts of interferon-*γ* (IFN-*γ*), induce delayed hypersensitivity, activate macrophages, and promote cellular immunity. Macrophages are an essential source of tumor necrosis factor-alpha (TNF-*α*) and play a crucial role in forming DKD [59]. A complete blockade of TNF-*α* expression was found in the diabetes-induced model after the knockdown of TNF-*α* in macrophages. In addition, macrophage TNF-*α* deletion caused decreased hypertrophy and proteinuria [60]. TGF-*β*1 secreted by macrophages regulates the expression of ferroptosis-related target genes ZEB1 and SLC7A11 by activating SMAD signaling transcription, and SLC7A11 gene deletion promotes iron overload-induced ferroptosis in macrophages [61, 62]. In other words, ferroptosis can be regulated by adjusting the expression of TGF-*β*1 in macrophages. This study confirms that PRDX6 and RGS4 are closely related to ferroptosis-mediated immune infiltration in DKD patients, especially RGS4. These results suggest that an imbalance between ferroptosis driver genes, suppressor genes, and marker genes has potential links to multiple underlying pathogenic factors in kidney disease, with immunity and inflammation as the main correlates, which are essential for finding multiple targets through ferroptosis interventions. Therefore, targeting ferroptosis-related immune cell responses, especially macrophages, is a potential strategy to improve the efficiency of immunotherapy in patients with DKD.

Therefore, we predict the small-molecule compounds kaempferol and quercetin associated with hub DE-FRGs and enrichment immune pathways and identify possible binding sites of proteins and ligands through molecular docking, thereby elucidating the relationship between drug and protein interactions at the molecular level. The relationship between drug molecules and protein interactions. The results showed that kaempferol and quercetin could also be applied to DUSP1, PRDX6, PEBP1, GABARAPL1, and RGS4 coding protein and play a role in treatment. Sharma et al. findings suggest that kaempferol inhibits hyperglycemia-induced activation of RhoA and decreases oxidative stress, proinflammatory cytokines (TNF-*α* and IL-1*β*), and fibrosis (TGF-*β*1 expression and extracellular matrix protein expression) in NRK-52E and RPTEC cells [63]. In vivo and in vitro studies showed that kaempferol treatment promoted the release of GLP-1 and insulin and increased the levels of cAMP and Ca2+ in GLUTag and MIN6 cells. It also increased GLP-1 and insulin release in the DKD mouse model. Kaempferol showed the potential to improve histological changes and renal fibrosis while reducing the expression levels of TGF-*β*1, CTGF, fibronectin, collagen IV, IL-1*β*, RhoA, ROCK2, and P-MYPT1 in DKD renal tissue [64]. Yuan et al. reported that kaempferol could activate nuclear factor-E2-related factor 2 (Nrf2)/SLC7A11/GPX4 signaling, enhance antioxidant capacity, inhibit the accumulation of lipid peroxidation in oxygen-glucose deprivation/reperfusion- (OGD/R-) treated neurons, and significantly reverse OGD/R-induced ferroptosis [65]. Quercetin (3,30,40,5,7-pentahydroxyflavone), a lipid-soluble compound, is also capable of radical scavenging, preventing lipid damage, and reducing lipid hydroperoxide production [66]. Studies have shown that quercetin can significantly reduce the expression of glycosylation end products, collagen IV, laminin, and connective tissue growth factor; inhibit the proliferation of mesangial cells (MC); and reduce the thickness of glomerular basement membrane [67, 68]. Quercetin can reduce iron content in pancreatic islets of diabetic mice. Although xCT is compensatorily upregulated, GSH and GPX4 are decreased in the DM model, which induces oxidative stress in pancreatic tissue, whereas quercetin can abrogate part of oxidative stress. Moreover, quercetin decreased the expression of the proapoptotic proteins Bax and cleaved-caspase 3, increased the expression of the antiapoptotic protein Bcl-2, and decreased the level of apoptosis induced by high glucose in mice and MP podocytes [69–71]. These results suggest that kaempferol and quercetin may exert immunotherapeutic effects on DKD by regulating ferroptosis.

This study provides a new direction for studying the pathogenesis of immune-related DKD. We first introduced the latest mechanism of ferroptosis in DKD, confirmed that ferroptosis modification is involved in the regulation of the immune microenvironment of DKD, and identified and validated a new ferroptosis score to predict the immune infiltration in DKD patients. In addition, we verified the docking binding sites of core compounds and core ferroptosis genes through molecular docking, providing guidance and suggestions for the development of new DKD drugs.

However, this study also has some shortcomings, which we must admit. Firstly, this study is based on bioinformatics analysis, and many results are theoretically valid but have not been verified experimentally, and their accuracy needs to be verified. The second is the sample size, especially in the combined analysis. Although the effect of interbatch differences is eliminated as much as possible, there are still other confounding factors that may affect our results. Third, the markers identified in this study associated with diagnosis and immunotherapy in patients with DKD are based on data from public databases, which we did not validate. In the future, we aim to design studies, conduct cell and animal experiments, and explore their possible biological mechanisms.

## 5. Conclusions

This study found that ferroptosis was associated with various pathogenic factors represented by immunity and inflammation in DKD. After accuracy analysis, seven hub ferroptosis genes (PRDX6, PEBP1, ZFP36, TSC22D3, GABARAPL1, and RGS4) could distinguish DKD and CON reliably. Based on immune infiltration analysis to evaluate the effect of ferroptosis on DKD, we found that hub ferroptosis genes and immune infiltration abundance were mainly negatively regulated, among which PRDX6 was significantly negatively regulated with immune cells. Meanwhile, RGS4 was significantly positively regulated with immune cell relationship deserves further study. At the same time, our scoring model of ferroptosis can reasonably predict the relationship between the clustering effect of ferroptosis and the abundance of immune cells. Finally, based on hub ferroptosis genes and enriched immune pathways, we predicted that Ren Shen and Huang Qi were potential drugs that could potentially affect ferroptosis to improve the immune and inflammatory mechanisms of DKD, with kaempferol and quercetin as the main active components.

## Figures and Tables

**Table 1:** Basic information of collected microarray datasets.

GEO	Platform	Tissue (Homo sapiens)	Samples (number)			Experiment type	PMID
			Total	CON	DKD		
GSE96804	GPL17586	Glomerulus	61	20	41	Expression profiling by array	30511699
GSE104948	GPL22945; GPL24120	Glomerulus	33	21	12	Expression profiling by array	297247043
GSE99339	GPL19109; GPL19184	Glomerulus	25	11	14	Expression profiling by array	28819298
GSE47183	GPL11670; GPL14663	Glomerulus	31	17	14	Expression profiling by array	24925724

**Table 2:** Molecular docking binding energy (kcal/mol).

Compound	Binding energy with PRDX6	Binding energy with PEBP1	Binding energy with GABARAPL1	Binding energy with RGS4	Binding energy with DUSP1
Kaempferol	-6.6	-8.3	-8.1	-8.5	-6.1
Quercetin	-6.5	-8.6	-8.3	-8.3	-6.0

## Data Availability

The data of this manuscript are all presented in the article.

## Supplementary Materials

Supplementary Materials
*Supplementary 1.* Figure S1: expression of DE-FRGs in the validation set. (a) Expression differences in validation set GSE99339. (b) Expression differences in validation set GSE47183. *Supplementary 2.* Figure S2: abundance of immune cell infiltration. (a) There were differences in immune cell infiltration between PRDX6 high expression group and low expression group in DKD. (b) There were differences in immune cell infiltration between RGS4 high expression group and low expression group in DKD. *Supplementary 3*. Figure S3: genetic clustering. (a) Cumulative distribution function (CDF) is displayed for *k* = 2–9. (b) The relative change in area under the CDF curve for *k* = 2–9. (c) The correlation between subgroups when cluster numbers *k* = 2. (d) Box plots of DE-FRG expression in gene clustering. (e) GSVA enrichment analysis of gene clustering. (f) Immune cell infiltration analysis based on gene clustering using ssGSEA algorithm. (g) Differential expression of immune checkpoints in gene clustering. (h) Differential expression of HLA genes in gene clustering. *Supplementary 4.* Figure S4: molecular docking model of the 2 key active ingredients with DUSP1. (a) The molecular docking of kaempferol with DUSP1 (PDB: 6APX). (b) The molecular docking of quercetin with DUSP1. *Supplementary 5.* Figure S5: molecular docking model of the 2 key active ingredients with DUSP1. (a) The molecular docking of kaempferol with PRDX6 (PDB: 1PRX). (b) The molecular docking of quercetin with PRDX6. *Supplementary 6.* Figure S6: molecular docking model of the 2 key active ingredients with DUSP1. (a) The molecular docking of kaempferol with GABARAPL1 (PDB: 2R2Q). (b) The molecular docking of quercetin with GABARAPL1. *Supplementary 7.* Table S1: prediction of ferroptosis and immune-related TCM.
