# Supplementary material for: Identification of Markers for Diagnosis and Treatment of Diabetic Kidney Disease Based on the Ferroptosis and Immune
Source: Oxid Med Cell Longev. 2022 Nov 23;2022:9957172. doi: 10.1155/2022/9957172 (PMC9712001; doi:10.1155/2022/9957172)
Supplement: Supplementary Materials — Supplementary 1. Figure S1: expression of DE-FRGs in the validation set. (a) Expression differences in validation set GSE99339. (b) Expression differences in validation set GSE47183. Supplementary 2. Figure S2: abundance of immune cell infiltration. (a) There were differences in immune cell infiltration between PRDX6 high expression group and low expression group in DKD. (b) There were differences in immune cell infiltration between RGS4 high expression group and low expression group in DKD. Supplementary 3. Figure S3: genetic clustering. (a) Cumulative distribution function (CDF) is displayed for k = 2–9. (b) The relative change in area under the CDF curve for k = 2–9. (c) The correlation between subgroups when cluster numbers k = 2. (d) Box plots of DE-FRG expression in gene clustering. (e) GSVA enrichment analysis of gene clustering. (f) Immune cell infiltration analysis based on gene clustering using ssGSEA algorithm. (g) Differential expression of immune checkpoints in gene clustering. (h) Differential expression of HLA genes in gene clustering. Supplementary 4. Figure S4: molecular docking model of the 2 key active ingredients with DUSP1. (a) The molecular docking of kaempferol with DUSP1 (PDB: 6APX). (b) The molecular docking of quercetin with DUSP1. Supplementary 5. Figure S5: molecular docking model of the 2 key active ingredients with DUSP1. (a) The molecular docking of kaempferol with PRDX6 (PDB: 1PRX). (b) The molecular docking of quercetin with PRDX6. Supplementary 6. Figure S6: molecular docking model of the 2 key active ingredients with DUSP1. (a) The molecular docking of kaempferol with GABARAPL1 (PDB: 2R2Q). (b) The molecular docking of quercetin with GABARAPL1. Supplementary 7. Table S1: prediction of ferroptosis and immune-related TCM. [file 9957172.f1.docx]

**
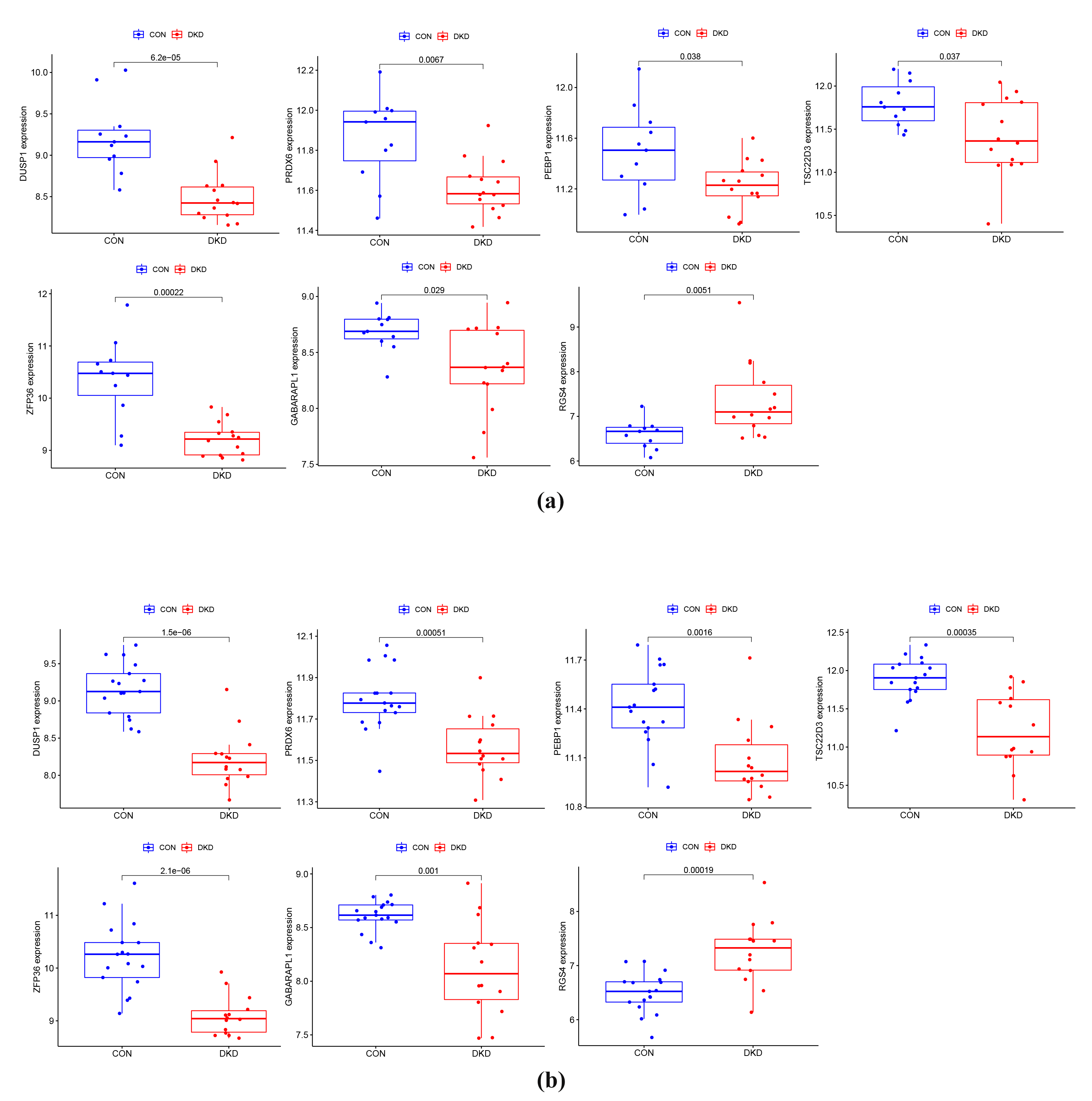
**

*Supplementary 1.* Figure S1:Expression of DE-FRGs in the validation set. (a)Expression differences in validation set GSE99339.(b)Expression differences in validation set GSE47183.

**
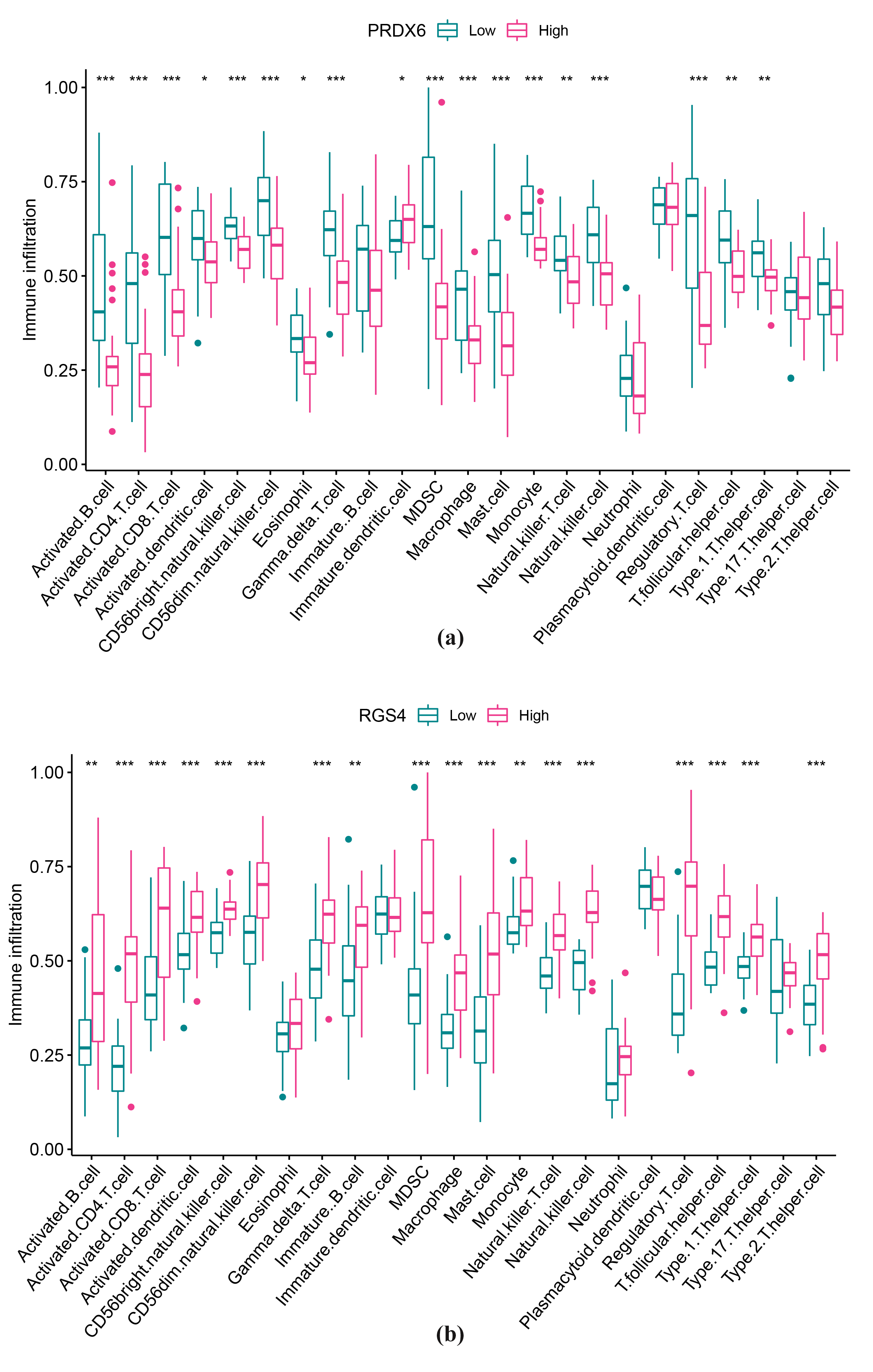
**

*Supplementary 2.* Figure S2:Abundance of immune cell infiltration. (a)There were differences in immune cell infiltration between PRDX6 high expression group and low expression group in DKD.(b)There were differences in immune cell infiltration between RGS4 high expression group and low expression group in DKD.

**
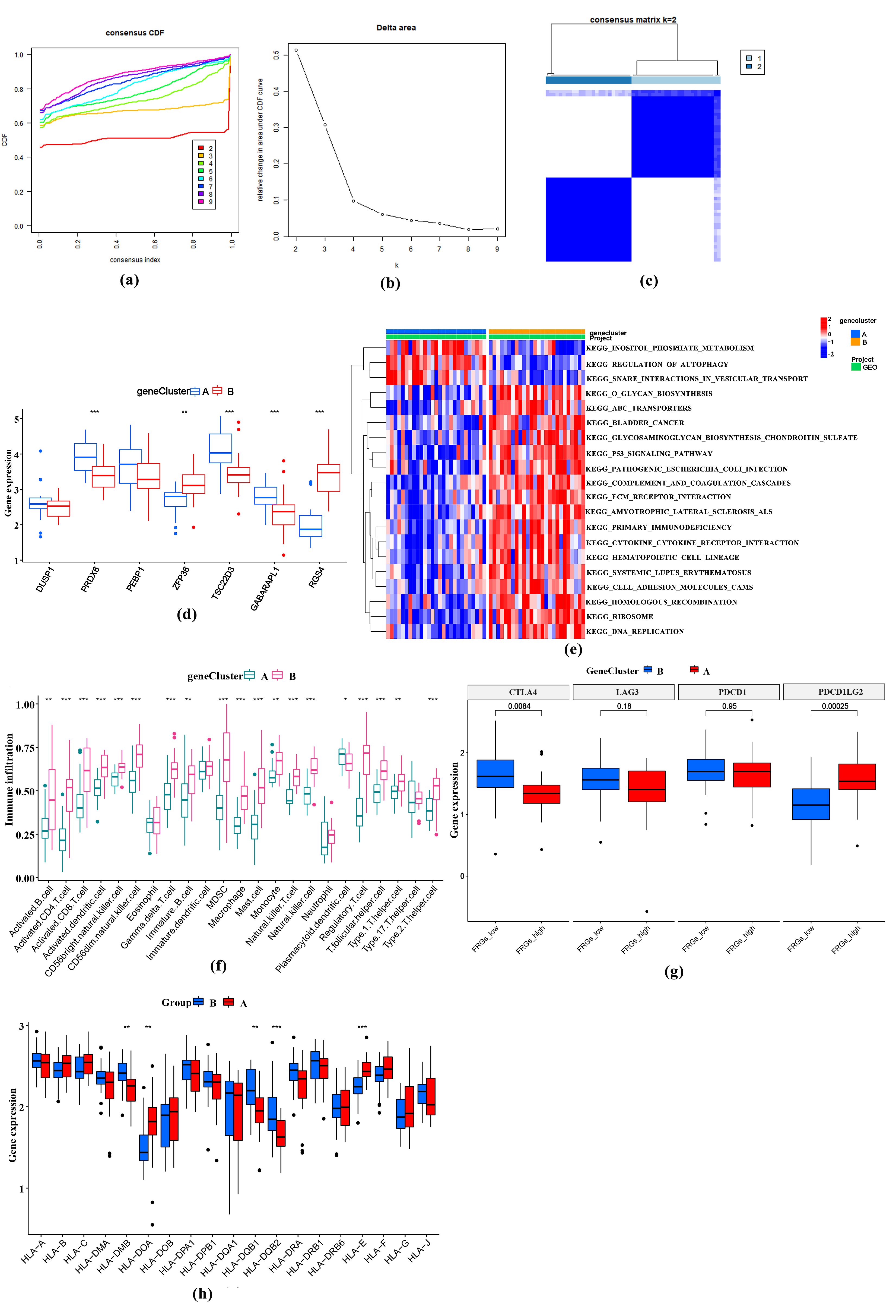
***Supplementary 3*. Figure S3:Genetic clustering.(a) Cumulative distribution function (CDF) is displayed for k = 2–9.(b) The relative change in area under the CDF curve for k = 2–9. (c)The correlation between subgroups when cluster numbers k = 2. (d) Boxplots of DE-FRGs expression in gene clustering.(e) GSVA enrichment analysis of gene clustering.(f) Immune cell infiltration analysis based on gene clustering using ssGSEA algorithm. (g) Differential expression of immune checkpoints in gene clustering.(h) Differential expression of HLA genes in gene clustering.

**
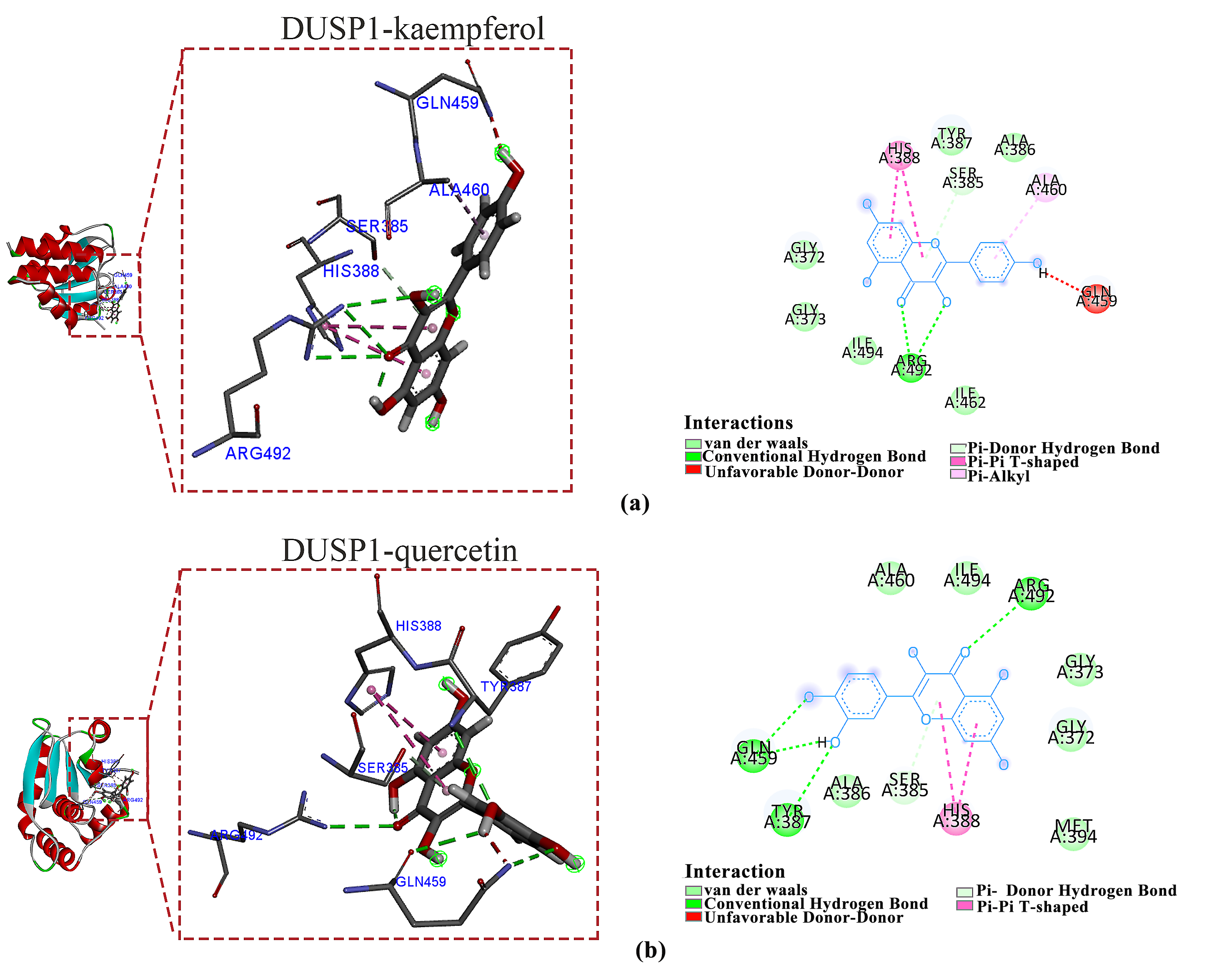
**

*Supplementary 4.* Figure S4:Molecular docking model of the 2 key active ingredients with DUSP1. (a)The molecular docking of kaempferol with DUSP1(PDB:6APX).(b)The molecular docking of quercetin with DUSP1.

**
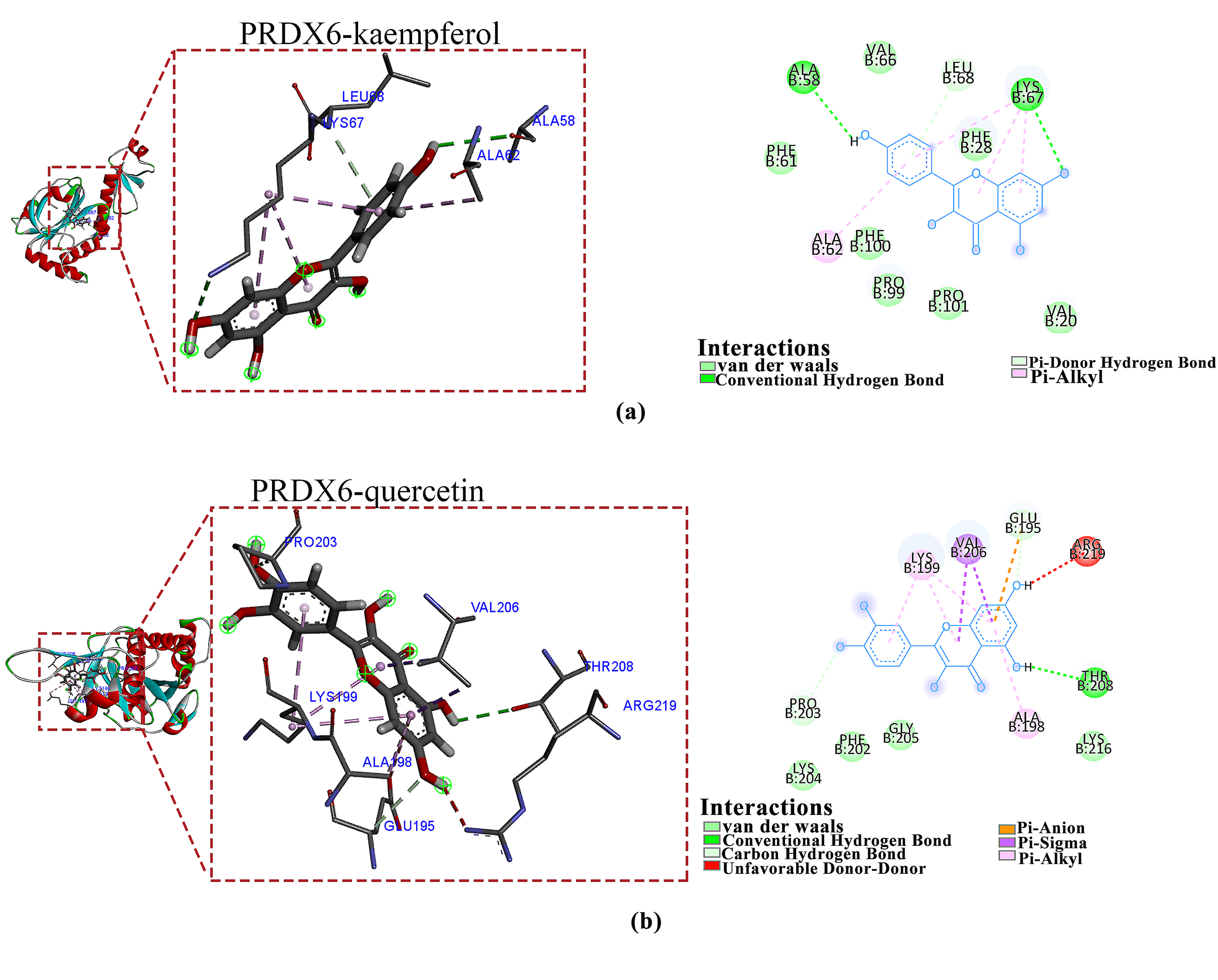
**

*Supplementary 5.* Figure S5:Molecular docking model of the 2 key active ingredients with DUSP1. (a)The molecular docking of kaempferol with PRDX6(PDB:1PRX).(b)The molecular docking of quercetin with PRDX6.

**
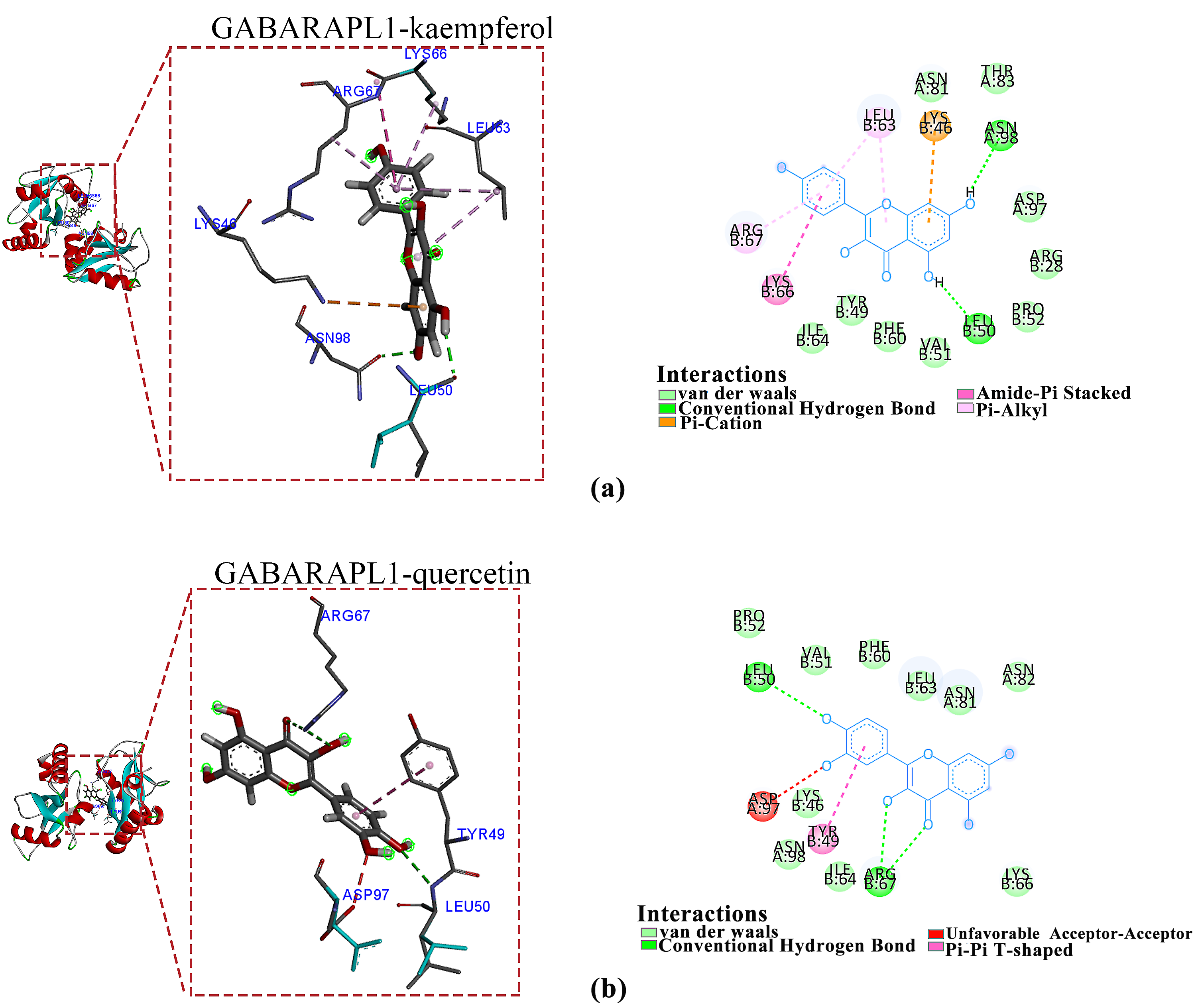
**

*Supplementary 6.* Figure S6:Molecular docking model of the 2 key active ingredients with DUSP1. (a)The molecular docking of kaempferol with GABARAPL1(PDB:2R2Q).(b)The molecular docking of quercetin with GABARAPL1.

*Supplementary 7.* Table S1: Prediction of ferroptosis and immune-related TCM.

| [Target](D:/ruanjian1/Dict/8.10.3.0/resultui/html/index.html#/javascript:;) [gene](D:/ruanjian1/Dict/8.10.3.0/resultui/html/index.html#/javascript:;) | Biological processes | Predict the TCM |
| --- | --- | --- |
| PRDX6 | T cell receptor signaling pathway | fang xie |
|  | natural killer cell mediated cytotoxicity | lou zi |
|  | B cell proliferation | shi di |
|  | B cell receptor signaling pathway | yu nao shi;xiao yu nao shi;yu biao jiao |
|  | T cell receptor signaling pathway | yu nao shi;xiao yu nao shi;yu biao jiao |
|  | B cell proliferation | shi ye |
|  | macrophage proliferation | sheng di huang |
|  | T cell activation | gan sui |
|  | T-helper 1 type immune response | yu nao shi;xiao yu nao shi;yu biao jiao |
|  | macrophage proliferation | di huang |
|  | T cell migration | yu nao shi;xiao yu nao shi;yu biao jiao |
|  | neutrophil degranulation | he geng;he ye ti;lian xu;shi lian zi;lian yi;ou jie;lian fang;Ou;he hua;he ye |
|  | neutrophil degranulation | lian zi xin;lian zi |
|  | T cell proliferation | shi di |
|  | humoral immune response | yu nao shi;xiao yu nao shi;yu biao jiao |
|  | T cell receptor signaling pathway | huang qin |
|  | T cell differentiation | huang qin |
|  | humoral immune response | shui niu jiao |
|  | natural killer cell mediated cytotoxicity | xiang hei zhong cao zi |
|  | macrophage activation | gan sui |
|  | T cell proliferation | shi ye |
|  | T-helper cell differentiation | dan shen |
|  | macrophage activation | he geng;he ye ti;lian xu;shi lian zi;lian yi;ou jie;lian fang;ou;he hua;he ye;lian zi xin;yu biao jiao;xiao yu nao shi;yu nao shi;lian zi |
|  | macrophage chemotaxis | dan shen |
|  | neutrophil chemotaxis | xiang hei zhong cao zi |
|  | T-helper 1 type immune response | yuan can e;can sha |
| PEBP1 | B cell proliferation | hong shen |
|  | T cell receptor signaling pathway | mang guo he |
|  | B cell proliferation | ren shen hua;ren shen lu;ren shen ye;ren shen |
|  | macrophage activation | ren shen hua;ren shen lu;ren shen ye;ren shen;huo shan shi hu |
|  | humoral immune response | shui niu jiao |
|  | natural killer cell mediated cytotoxicit | ren shen hua;ren shen lu;ren shen ye;ren shen |
|  | T-helper cell differentiation | ren shen;hua;ren shen lu;ren shen ye;ren shen |
|  | macrophage activation | jiang pi;sheng jiang;gan jiang;yang shen ye  ;xi yang shen;hong shen |
|  | regulatory T cell differentiation | ren shen hua;ren shen lu;ren shen ye;ren shen |
|  | T-helper 1 type immune response | ren shen hua;ren shen lu;ren shen ye;ren shen |
|  | granulocyte macrophage colony-stimulating factor production | ren shen hua;ren shen lu;ren shen ye;ren shen |
|  | B cell activation | gou shu pi |
|  | humoral immune response | hong shen |
|  | T cell differentiation | mang guo he |
| GABARAPL1 | T-helper 1 type immune response | yuan can e;can sha |
|  |  |  |
|  | macrophage activation | dong chong xia cao |
|  | T cell activation | gan sui |
|  | humoral immune response | yuan can e;can sha |
|  |  |  |
|  | macrophage activation | gan sui |
|  | B cell activation | mei hua;wu mei |
|  |  |  |
|  | T cell proliferation | yuan can e;can sha |
|  |  |  |
| DUSP1 | mononuclear cell migration | zi cao |
|  | macrophage chemotaxis | zi cao |
|  | monocyte chemotaxis | zi cao |
|  | humoral immune response | yan jing she |
|  | T cell proliferation | gou qi zi |
|  | T cell mediated cytotoxicity | gou qi zi |
|  | B cell proliferation | jiao gu lan |
|  | macrophage activation | mu xiang |
|  | B cell proliferation | gou qi zi |
|  | T-helper cell differentiation | huang si yu jin |
|  | T-helper cell differentiation | jiang huang |
|  | T-helper 1 type immune response | gou qi zi |
|  | B cell proliferation | mo jia huang qi |
|  | T cell differentiation | huang qin |
|  | humoral immune response | shui niu jiao |
|  | T cell receptor signaling pathway | huang qin |
|  | T-helper 1 type immune response | mo jia huang qi |
|  | macrophage proliferation1 | huang si yu jin;jiang huang |
|  |  |  |
|  | macrophage activation | mo jia huang qi |
|  | T cell proliferation | mo jia huang qi |
|  | humoral immune response | huang si yu jin;jiang huang |
|  |  |  |
|  | macrophage activation | yan jing she |
|  | humoral immune response | mu xiang |
| RGS4 | T cell receptor signaling pathway | yuan she xiang |
|  | B cell activation | yuan she xiang |
| ZFP36 | T cell migration | sang shen;sang bai pi |
|  |  |  |
|  | T cell differentiation | rou gui |
|  | T cell migration | sang zhi;sang ye |
|  |  |  |
|  | B cell activation | gou shu pi |
|  | macrophage activation | rou gui |
|  | T cell proliferation | rou gui |
